# A New Interpreter of Faith

**Published:** 1891-03-28

**Authors:** 


### THE DOCTOR'S ARMCHAIR.

A NEW INTERPRETER OF FAITH.

THERE are those who hold that a new religion is needed if the world is to be saved from atheism. There are others who prefer atheism to religion. To neither class does Sir Andrew Clark, the President of the Royal College of Physicians, belong. But he seems to be very certain that the boundaries of faith require to be enlarged, and its essence to be better understood. He believes, if we understand him aright, that the religious man of science has arrived at a truer appreciation of faith than the religious ecclesiastic or theologian. The following may be taken as a somewhat expanded definition of faith according to this representative physician: "Faith," says Sir Andrew Clark, "is assurance or certitude, supported by knowledge and reason, adjusted to the development of the higher nature of man, and waiting for demonstration. . . It is a fundamental faculty or function of the human organism whereby, in certain conditions, we recognise not only our organic relations with God, but also amidst the manifold things which surround and pervade us, the things which are His and the way in which we are to walk amongst them. . . Almost all thoughtful Christians are now agreed that faith rests neither on the Bible nor on the Church, neither on councils nor on authority of any kind, but upon the inspiration of God acting through the faculty which He has given to man."

There are two points here of great moment: the one suggests a problem of profound interest to the man of science, and of great practical importance to all men. The other pulls up short both the ecclesiastic and the theologian, and demands that they shall give a scientific reason for their position. Is it true, as Sir Andrew Clark affirms, that faith is a fundamental or primitive faculty of the human organism? It is a pity that the speaker, when he affirmed this remarkable utterance before the Church Congress in October last did not make some clear and comprehensive effort at convincing demonstration. "Faith is a primitive faculty of the human organism:" it is, we assume Sir Andrew to mean, peculiar to the human organism; it is not a primitive faculty of the lower animals; it is a differentiating mark which separates man by an impassable gulf from all the animals which do not belong to the genus man. This, it seems to us, is the logical corollary which follows upon Sir Andrew's hypothesis; and it is exactly here where the forces of faith and non-faith will join issue.

To the ordinary evolutionist the convincing demonstration of Sir Andrew's position would be a blow which would shatter his whole fabric to pieces. Sir Andrew himself seems to perceive this, although he holds that his affirmation does not necessarily demolish the evolutionary theory as regards animals, but only as regards man. Here, it seems to us, Sir Andrew's logic very decidedly halts. But the main point at issue is the demonstrableness or otherwise of the actual position affirmed. Is it true or is it not true that faith is a primitive faculty of the human organism? How shall we set to work to make the demonstration? If we are wise we shall spin no *à priori* theories. The only rational course to take is to get hard down upon the facts. Observation, cold, passionless, and unprejudiced, is the electric candle which will light us to the truth.

But what is faith? We want a more precise definition before we can proceed. We are going to look among the primitive faculties of man for a particular object. What is that object? What is it like? How shall we recognise it? Does it ever change its form? Does it perform always one and the same function, or a variety of functions? And does it assume different shapes for its different functions? Has it any constant and unvarying marks whereby it may infallibly be recognised when seen? And if it has, what are those marks?

A definition, it is evident, must be at least attempted before another step can be taken. Faith is that faculty which acts upon the assumption that a certain result will follow, whether the result has been previously demonstrated to the reason or not. This means, of course, that faith sometimes acts at the bidding of reason, and sometimes at the bidding of an inner impulsion, of which it may be conscious or unconscious, but which it cannot always explain. According to this definition faith may exist and be active in a man whose reason is fully developed; it may also exist and be active in a child who has not yet reached the period when reason is apparent. If faith be a "primitive faculty of the human organism," it must be present in the child; and if adult reasoners are to recognise it as a primitive faculty, they must be able to perceive it in the child. The first part of the demonstration then must begin with the newly born.

Has the newly-born infant any faith? The thinking man who stands by the bedside of the mother with her newly-born babe does not incline to put the question that way. After seeing the actual behaviour of the child with the mother he feels rather that he must ask not whether the child has any faith, but whether it has anything else but faith? Does not the child, indeed, in the most literal sense, "live by faith"? This much, at any rate, is certain, that the child performs certain definite actions. Are those actions altogether reflex, or are they, from the first, partly voluntary; and does the voluntary element tend to increase and the reflex to diminish with increasing age? The actions of the newly-born, from the earliest day, show undoubtedly a voluntary element; and when the child, of its own impulsion, seeks the mother's breast, it performs an act of faith.

The hasty reasoner rushes in here, and declares that it is "instinct," not faith, which impels the child to action. But to describe the act as one of "instinct" rather than one of "faith" is not to change the character of the act in the least degree: it is only to give it another name. Suppose we substitute for "faith" or "instinct" the word "trust," we shall then put a label upon the child's act which practically everybody will agree to call descriptive. When the child seeks his mother's breast he performs an act of "trust." But what is "trust" if it be not "faith"?

As the child advances in age, and intelligence is developed, do we find that faith increases or diminishes? We cannot, perhaps, say that it increases, but in normal circumstances it certainly does not tend to diminish.

What really happens is this: reason becomes increasingly active; and whilst reason may add but little strength to faith, it undoubtedly adds a great deal of pleasure, a pleasure of which the child becomes day by day increasingly conscious. Childhood, it cannot be denied, displays faith as a primitive faculty. It may, indeed, be affirmed that of all the primitive faculties of the child's mind, faith is not only the strongest but the most conspicuous and the most constant. Of what value this particular fact may be to Sir Andrew Clark's arguments we are not prepared to say offhand, because this kind of faith is not at all peculiar to the human organism, but is shared with man by all the rest of the vertebrate creation, and undoubtedly by a large part of the invertebrate.

It remains to follow up the path of observation from infancy to childhood, from childhood to youth, and from youth to manhood. But that we have no space for in the present paper. It probably cannot philosophically be denied that faith is a primitive faculty of the human organism. But as little can it be denied that faith is a primitive faculty of all organisms that are endowed with any measure of volition. Religious faith is something more than the mere primitive trust of the child. If it is not it is what we call a "superstition"; a superstition in that it is not guided, informed, and regulated by the reason. The primitive faculty has still to ask the reason to take it by the hand, and the faith and the reason together have to look to the objective phenomena of the world for help and suggestion; and these objective phenomena give hints of a cause that preceded and produced them. As Paul said, "The invisible things of God from the foundation of the world are clearly seen, being understood by the things that are made; even His eternal power and Godhead."

But when faith and reason, walking hand in hand, have finished their investigations of the objective phenomena—are they satisfied? Is either of them satisfied? Not in the least! Each of them is still in the condition of a man who wanders at midnight on a lonely moor. Light he asks, and light he must have! The very fact that of all the twelve hundred millions of the world's inhabitants, not twelve hundred thousand disbelieve in a Divine revelation is the most convincing circumstance in the world that both primitive faith and primitive reason count themselves to have completely failed if they do not succeed in finding something which they believe to be a Divine revelation for a final resting-place. Here we must stop. Sir Andrew Clark has let down his plumb line into a deep well; we venture to urge upon him that he will continue his soundings, and let the world hear of his results.

